# ^18^F-FLT and ^18^F-FDG PET/CT in Predicting Response to Chemoradiotherapy in Nasopharyngeal Carcinoma: Preliminary Results

**DOI:** 10.1038/srep40552

**Published:** 2017-01-16

**Authors:** Shi Qi, Yang Zhongyi, Zhang Yingjian, Hu Chaosu

**Affiliations:** 1 Department of Radiation Oncology, Fudan University Shanghai Cancer Center, Department of Oncology, Shanghai Medical College, Fudan University, Shanghai, 200032, China; 2 Department of Nuclear Medicine, Fudan University Shanghai Cancer Center; Department of Oncology, Shanghai Medical College, Fudan University; Center for Biomedical Imaging, Fudan University; Shanghai Engineering Research Center for Molecular Imaging Probes, Shanghai 200032, China

## Abstract

The purpose of this study was to explore the feasibility of ^18^F-Fluorothymidine (^18^F-FLT) and ^18^F-Fluorodeoxyglucose (^18^F-FDG) positron emission tomography/computed tomography (PET/CT) in predicting treatment response of nasopharyngeal carcinoma (NPC). Patients with NPC of Stage II-IVB were prospectively enrolled, receiving 2 cycles of neoadjuvant chemotherapy (NACT), followed by concurrent chemoradiotherapy. Each patient underwent pretreatment and post-NACT FLT PET/CT and FDG PET/CT. Standard uptake values (SUV) and tumor volume were measured. Tumor response to NACT was evaluated before radiotherapy by MRI (magnetic resonance imaging), and tumor regression at the end of radiotherapy was evaluated at 55 Gy, according to RECIST 1.1 Criteria. Finally, 20 patients were consecutively enrolled. At the end of radiotherapy, 7 patients reached complete regression while others were partial regression. After 2 cycles of NACT both FLT and FDG parameters declined remarkably. Parameters of FDG PET were more strongly correlated to tumor regression than those of FLT PET.70% SUVmax was the best threshold to define contouring margin around the target. Some residual lesions after NACT showed by MRI were negative in PET/CT. Preliminary results showed both ^18^F-FDG and ^18^F-FLT PET have the potential to monitor and predict tumor regression.

While nasopharyngeal carcinoma (NPC) is a rare malignant tumor, it is one of the most endemic head and neck cancers in southern China, Southeast Asia, the Middle East, North Africa, Alaska, Greenland, and the Mediterranean^1^. Meta-analysis has confirmed the benefits associated with the addition of chemotherapy to radiotherapy in nasopharyngeal carcinoma^2^, especially for local-regionally advanced cases^3^,^4^,^5^. Nevertheless, non-CR (complete regression) cases still exist and these patients may have a poorer prognosis including overall, failure-free and distant metastasis-free survival^6^. Identification of prognostic factors which predict tumor and lymph nodes response may guide individualized treatment better, and thus improve prognosis.

^18^F-Fluorodeoxyglucose positron emission tomography (^18^F-FDG PET) is now commonly used in clinical diagnosis and in assisting tumor staging of NPC. It is not only a tool showing tumor images, but also one of the new tools that can capture tumor biology without an invasive procedure^7^. Although the value of ^18^F-FDG PET comparing with computed tomography (CT) and magnetic resonance imaging (MRI) in diagnosing head and neck cancer is still an open question^8^,^9^,^10^,^11^,^12^, its sensitivity and specificity in detecting distant metastasis is higher^13^,^14^,^15^,^16^,^17^. As its capability in revealing biological feature of tumor, researchers have become interested in the predictive value of FDG PET, including for prognosis and for tumor regression of NPC^18^,^19^,^20^.

Intrinsic radiosensitivity and tumor repopulation (along with cell repair, re-oxygenation and cell cycle redistribution) are two of the five “R”s determining radiotherapy efficacy according to classical radiobiology^21^. Tumor proliferation is a factor closely related to both the two “R”s. Methods monitoring tumor proliferation may have potential in predicting tumor response and prognosis. ^18^F-Fluorothymidine (FLT) PET is a non-invasive measurement evaluating tumor proliferation. After intravenous injection, the intracellular trapping of ^18^F-FLT occurs through phosphorylation by thymidine kinase 1. Thymidine kinase 1 is a key enzyme in DNA synthesis, with high enzymatic activity observed during the S phase of the cell cycle and low activity in the G0/G1 phase^22^. Thus, accumulation of ^18^F-FLT is considered an indirect marker of active cellular proliferation. It has been shown that ^18^F-FLT uptake strongly correlates with the proliferation index as measured by Ki-67 immunohistochemistry in lung and breast tumors^23^. ^18^F-FLT uptake has been reported in untreated squamous cell head and neck cancers^24^,^25^,^26^,^27^. Its potential to predict radiosensitivity has been validated in NPC xenografts nude mice model^28^. However, there are no reports on its predictive value in NPC patients.

The main purpose of this study is to explore the feasibility of FLT PET and FDG PET in monitoring and predicting treatment response of NPC, and to compare their efficiency as well.

## Materials and Methods

### Ethical Approval and Informed Consent

All procedures performed in studies involving human participants were in accordance with the ethical standards of Fudan University Shanghai Cancer Center Ethics Committee and with the 1964 Helsinki declaration and its later amendments or comparable ethical standards. The experimental protocol was approved by both Institutional Review Board and Ethics Committee of Fudan University Shanghai Cancer Center. The methods were carried out in accordance with the relevant guidelines and regulations. Informed consent was obtained from all individual participants included in the study.

### Patients and Pretreatment Evaluation

The inclusion criteria were as following: (1) biopsy-proven primary nasopharyngeal carcinoma; WHO type II or type III; (2) T3-4N0-3M0 and T1-2N2-3M0 according to 2010 UICC/AJCC (Union for International Cancer Control/American Joint Committee on Cancer) staging system; (3) from 18 to 70 years old; (4) pretreatment ANC (absolute neutrophil count) > 2.0 × 10^9^/L, Platelet > 100 × 10^9^/L; TB (total bilirubin) < 34.2 μmol/L, ALT (alanine aminotransferase) < two fold upper limit of reference value, blood creatinine < 1.5 mg/dL, CCr (creatinine clearance rate) > 50 ml/min; (5) All patients provide written informed consent to participate in the study. The exclusion criteria were as follows: (1) with distant metastasis proven by clinical or radiologic evidence; (2) with history of radiation or surgery against head and neck cancer, or history of chemotherapy in one month; (3) with any other primary malignant tumor previously diagnosed, or combined concurrently; (4) with diabetes mellitus diagnosed; (5) women of childbearing age whose result of pregnancy test is positive; (6) with psychiatric disorders or other mental diseases affecting the capability of understanding and signing informed consent.

MRI scans of nasopharynx and neck, and whole-body ^18^F-FLT and ^18^F-FDG PET/CT should be completed in two weeks before treatment, and should be repeated after 2 cycles of neoadjuvant chemotherapy (NACT).

### PET/CT Imaging

The interval between ^18^F-FLT and ^18^F-FDG PET/CT was less than 3 days. ^18^F-FLT was prepared according to our published method^29^. ^18^F-FDG was produced automatically by cyclotron (Siemens CTI RDS Eclips ST, Knoxville, Tennessee, USA) using Explora FDG_4_ module in our center. Radiochemical purities of them were both over 95%.

^18^F-FLT and ^18^F-FDG PET/CT studies were performed on 2 separate days. Before the ^18^F-FDG PET/CT, all patients were requested to fast at least 4 h. At the time of tracer injection (dosage: 7.4 MBq/kg), patients presented blood glucose level under 10 mmol/L. Before and after injection, patients were kept lying comfortably in a quiet, dimly lit room. Scanning was initiated 1 hour after administration of tracer. Images were obtained on a Siemens biograph 16HR PET/CT scanner (Knoxville, Tennessee, USA). Transaxial intrinsic spatial resolution was 4.1 mm (full-width at half-maximum) in the center of the field of view. Data acquisition procedure was as follows: CT scanning was first performed, from the proximal thighs to head, with 120 kV, 80 ~ 250 mA, pitch 3.6, rotation time 0.5. Immediately after CT scanning, a PET emission scan that covered the identical transverse field of view was obtained. Acquisition time was 2 ~ 3 min per table position. PET image data sets were reconstructed iteratively by applying CT data for attenuation correction, and coregistered images were displayed on a workstation.

^18^F-FLT PET/CT imaging: All conditions were the same as ^18^F-FDG PET/CT imaging, except that blood glucose control and movement restrictions before imaging were not required.

### PET Imaging interpretation

A multimodality computer platform (Syngo, Siemens, Knoxville, Tennessee, USA) was used for image review and manipulation. Two experienced nuclear medicine physician evaluated the images independently. The reviewers reached a consensus in cases of discrepancy.

Glucose metabolic activity and proliferative activity were quantified using the Standardized Uptake Value (SUV) normalized to body weight. Maximal and mean SUV for primary tumor (SUVmax-T, SUVmean-T) and lymph nodes (SUVmax-N, SUVmean-N), MTV (metabolic tumor volume) and PTV (proliferative tumor volume) were calculated. Boundaries were drawn large enough to include all lesions in axial, coronal, and sagittal PET images. To define the contouring margins, we tried 40%, 50%, 60 and 70% SUVmax for both FDG and FLT PET, and tried SUV of 2.5 for FDG PET and 1.5 for FLT PET as threshold. TLG (total lesion glucose) and TLT (total lesion thymidine) were calculated according to the formula: TLG (TLT = SUVmean × MTV (PTV). Σparameter were calculated as the sum of parameters of all lesions. The percent of each Σparameter declined after 2 cycles of NACT was recorded as Per-parameter, for example Per-SUVmax.

### Radiotherapy

#### Intensity-Modulated Radiation Therapy (IMRT)

IMRT was applied to all patients enrolled. The total dose to primary tumor was 66 Gy in 30 fractions for T1 or T2 disease, 70.4 Gy in 32 fractions for T3 or T4 lesion. A total dose of 60 Gy was delivered to the CTV in 30–32 fractions.

### Chemotherapy

NACT was consisted of docetaxel 75 mg/m^2^ IV on day 1, cisplatin 75 mg/m^2^ IV on day 1, and 5-fu 500 mg/m^2^ d continuously IV on day1–5. This was followed by cisplatin 80 mg/m^2^ IV every three week during radiation.

### Response evaluation

MRI at the end of radiotherapy was conducted at 55 Gy. Tumor regression was evaluated by MRI according to RECIST (Response Evaluation Criteria in Solid Tumors) 1.1 Criteria. Late toxicities were evaluated according to the toxicity criteria of the RTOG^30^.

### Statistical analysis

All analyses were performed using SPSS, version 19.0. The primary endpoint was tumor regression, which was defined by RECIST 1.1. Correlation between tumor regression (both at post-NACT and the end of radiotherapy) and PET parameters was estimated by Spearman Rank Test. Difference between corresponding PET parameters was tested by two-tailed t test.

## Results

### Patient characteristics and treatment outcome

Since November 2012, 20 patients have been consecutively enrolled into this prospective study. The median follow-up period was 39 months. Patient characteristics are listed in Table 1. Pathologic types of all patients were non-keratinizing, undifferentiated. Evaluated with MRI after NACT, only one patient reached complete regression (CR), while all other patients reached partial regression (PR). This female patient reaching CR after NACT had a stage of T2N2M0 at baseline. At the end of radiotherapy (55 Gy), 7 patients reached CR while others still stayed at PR. So far, loco-regional recurrence or distant metastasis has occurred to none of those patients.

### Baseline FLT and FDG PET

The diagnostic sensitivity of both FLT and FDG PET before NACT were 100%. All primary lesion and lymph nodes showed in FDG PET could be visualized in FLT PET. In FLT PET, the median SUVmax of primary tumor and of lymph nodes were 5.97, ranging from 3.12–12.48, and 6.66, ranging from 3.52–10.5. In FDG PET, the median SUVmax of primary tumor and of lymph nodes were 8.97, ranging from 5.20–19.78, and 12.42, ranging from 3.73–25.10. Compared with FDG PET, maximal uptake value of FLT PET at baseline was significantly lower (Table 2). In spite of this, all corresponding parameters of FLT and FDG PET were moderately or strongly related to each other, no matter which threshold we used to define the contouring margins around the target.

### Evaluating tumor response with FLT and FDG PET

**Figure 1:**
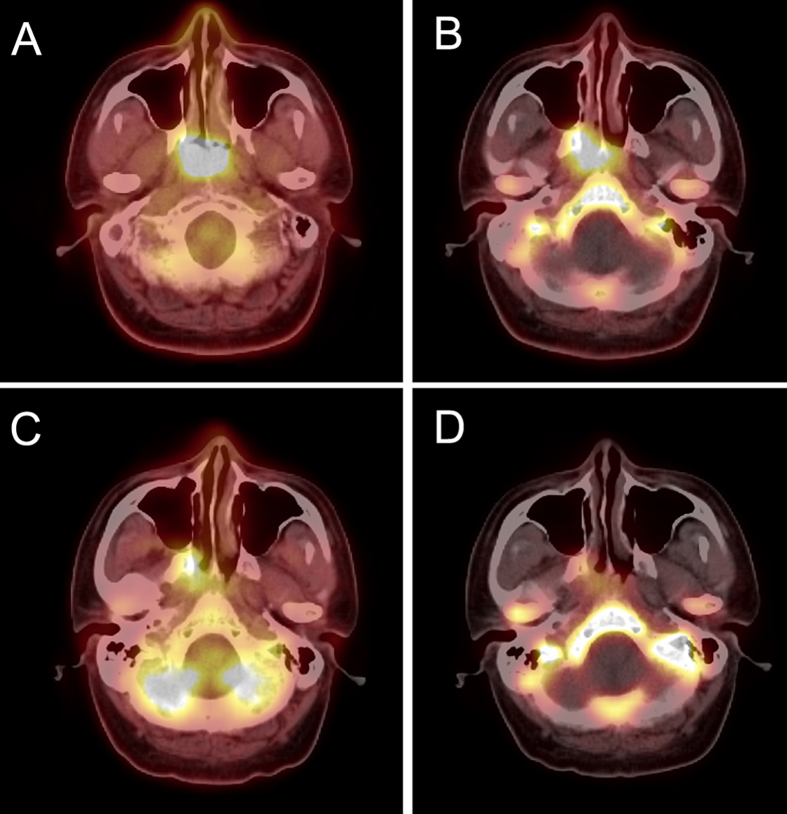
A 36-year-old male NPC patient, we detected high FDG (**A**) and FLT (**B**) accumulations in primary lesion, with the SUVmax of 13.1 and 6.5, respectively. We still find obvious uptake of both FDG (**C**) and FLT (**D**) after two cycles of NACT, SUVmax = 6.6 and 4.9. At the end of radiotherapy (55 Gy), he reached only PR according to RECIST criteria.

**Figure 2:**
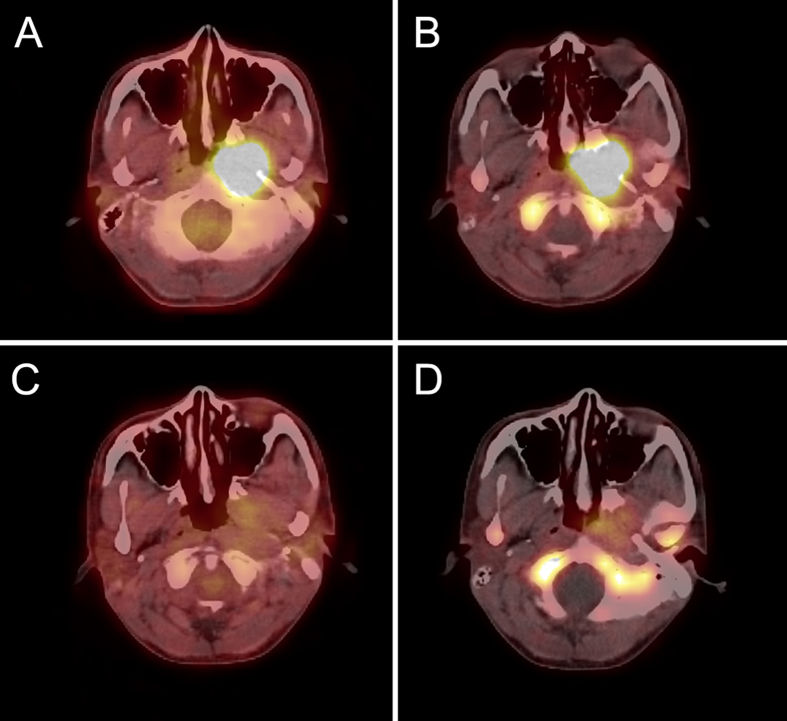
A 22-year-old male NPC patient, pre-treatment PET/CT showed high FDG (**A**) and FLT (**B**) uptake in primary lesion, SUVmax = 9.5 and 8.9, respectively. However, after two cycles of NACT, only mild FDG (**C**) and FLT (**D**) uptake was detected, SUVmax = 2.1 and 2.9. At the end of radiotherapy, it was diagnosed CR.

After 2 cycles of NACT, both FLT and FDG parameters declined remarkably. Figures 1 and 2 are images of our 2 patients, including FLT and FDG PET/CT images before and after 2 cycles of NACT. Baseline ΣSUVmax of FLT PET was 31.01 ± 11.92, which decreased significantly to 9.54 ± 8.29 after induction chemotherapy (p = 0.000). Similarly, baseline ΣSUVmax of FDG PET was 48.50 ± 18.65, which decreased significantly to 11.36 ± 8.23 after induction chemotherapy (p = 0.000).

**Figure 3:**
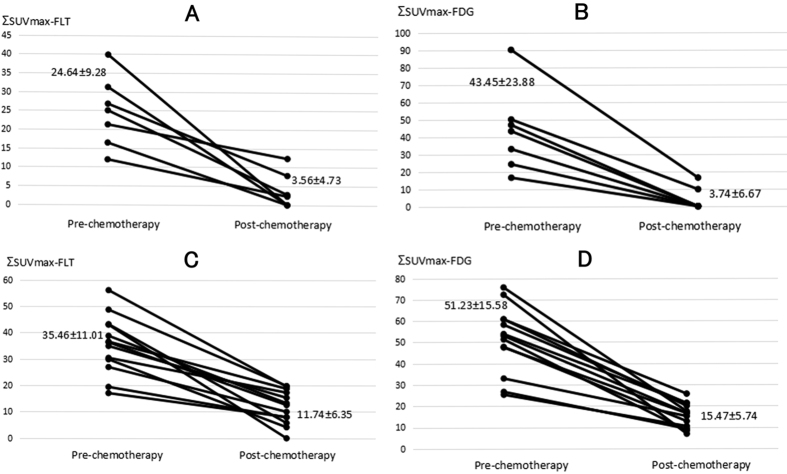
In the subgroups of CR (complete regression) patients, ΣSUVmax of FLT (Fig. 3A) and FDG (Fig. 3B) PET decreased dramatically after NACT (p = 0.003, p = 0.001). In the subgroups of PR (partial regression) patients, ΣSUVmax of FLT (Fig. 3A) and FDG (Fig. 3B) PET decreased dramatically after NACT (p = 0.000, p = 0.000).

In both subgroups of CR and PR patients, ΣSUVmax of FLT and FDG PET decreased dramatically after NACT (Fig. 3). In the subgroup of 7 CR patients, although the baseline ΣSUVmax of FLT and FDG PET was lower (24.64 ± 9.28, 43.45 ± 23.88) than those of the subgroup of 13 PR patients (35.46 ± 11.01, 51.23 ± 15.58), statistical differences was only detected in FLT (p = 0.041 and 0.388). Nevertheless, post-NACT ΣSUVmax of FLT and FDG PET showed significant difference between CR and PR groups (FLT: 3.58 ± 4.73 and 11.74 ± 6.35, p = 0.008; FDG: 3.74 ± 6.67 and 15.47 ± 5.74, p = 0.001). Additionally, Per-SUVmax of FLT between CR and PR groups were 0.86 ± 0.21 and 0.56 ± 0.42 (p = 0.074); and those of FDG were 0.95 ± 0.09 and 0.68 ± 0.11 (p = 0.000). FDG PET might show higher predictive value than FLT PET in differentiating CR and PR.

### Correlation between PET Parameters and Tumor Regression

As only one patient achieved CR while others achieved PR after NACT, none of pre-NACT PET parameter was correlated with post-NACT tumor regression.

As for the point at the end of radiotherapy, none of pre-NACT PET parameter was correlated with tumor regression; however, moderate or strong correlations were revealed between post-NACT PET parameters and tumor regression (Table 3). According to Table 3, parameters of FDG PET were more strongly correlated to tumor regression than those of FLT PET in generally. In both FLT and FDG PET, threshold of 70% SUVmax was better than others, according to the correlation index.

### The comparison of predictive value between MRI and PET

MRI diagnosed one patient achieved CR while others were all PR after 2 cycles of NACT; therefore, correlation between post-NACT tumor regression assessed by MRI and tumor regression at the end of radiotherapy was too poor. As for PET, 4 patients were evaluated as CR by FLT PET and 5 were evaluated as CR by FDG PET after NACT. Therefore, some residual lesions after NACT showed by MRI were negative in PET/CT. Both FLT and FDG PET could reflect the metabolic changes of tumor and predict the prognosis more early than MRI.

## Discussion

The present study tested the potential of monitoring and predicting treatment efficiency with FLT and FDG PET in locoregionally advanced nasopharyngeal carcinoma. The preliminary results reported that after 2 cycles of NACT, parameters of FDG and FLT PET decreased significantly, and revealed that Parameters of both FDG and FLT PET (ΣSUVmax, Per-SUVmax, ΣSUVmean, Per-Suvmean, ΣPTV, Per-PTV, ΣTLT, Per-TLT, ΣMTV, Per-MTV, ΣTLG, Per-TLG) had medium to strong correlation with tumor response after chemoradiotherapy.

In recent years, the value of FDG PET was not restricted to diagnosis and detecting metastatic lesions in NPC. Its predictive value in prognosis of locally advanced NPC has been revealed. SUV, MTV and heterogeneity index (HI = SUVmax/SUVmean) might have the potential to predict long-term outcome including LC (local recurrence), PFS (progression-free suvival) and OS (overall survival)^18^,^19^,^20^. Thus, we hypothesized that FDG PET may have the capability in predicting tumor regression of NPC after radiotherapy as well. FLT PET is a new diagnostic technique evaluating tumor proliferation. Its value in reporting and predicting tumor response to radiation or chemotherapy had attracted a lot of interest. There have been several studies of other malignant tumors using FLT PET in early prediction or monitoring of tumor response to chemotherapy or chemoradiotherapy. Trigonis *et al*. reported that RT induced an early, significant decrease in lesion FLT uptake of NSCLC (non-squamous cell lung carcinoma), which confirm the potential for FLT PET to report early on radiation response^31^. Park *et al*. suggested that the percent change of tumor uptake in ^18^F-FLT PET after induction chemotherapy might be feasible for early prediction of tumor response after induction chemo-therapy and concurrent chemoradiotherapy in patients with esophageal cancer^32^. In head and neck cancer, in addition to studies of kinetic analysis and of its diagnostic value^24^,^26^,^27^, there also have been studies focusing on the prognosis-predictive value of FLT PET. Linecker *et al*. reported that ^18^F-FLT uptake is inversely correlated with patient survival, as well as ^18^F-FDG, although FLT does not provide additional visual information in comparison to FDG^25^. In NPC, there has been no published clinical study exploring predictive value of FLT PET for radiosensitivity and prognosis. The result of our study suggested that FLT PET has the capability of monitoring early tumor response after NACT and may have the potential to predict radiosensitivity for its correlationship with tumor regrssion after concurrent chemoradiotherapy, as well as FDG PET.

70% SUVmax was potentially the best threshold for both FLT and FDG PET. Although SUVmax of 2.5 was commonly used as a diagnostic threshold for malignancy in FDG PET, researchers have realized that a common threshold does not exist. SUVmax thresholds may vary depending on the metabolic activity of the lesions but also on the prevalence of inflammation and infection in a specific region of the body. Interpretation of FDG PET is usually based on visual evaluation and not on SUV measurements because data have shown that the use of SUV failed to be more accurate than the visual evaluation in predicting the presence of malignanc^33^,^34^,^35^. A study focusing on head and neck cancer (HNC) showed the sSUV (suitable standard uptake value) had a linear correlation with the SUVmax, which indicated that for HNC, a suitable threshold or SUV level can be established by correlating with SUVmax rather than using a fixed SUVmax^36^. In this study, as the diagnostic sensitivity of both PET techniques were 100%, whichever threshold was used; an important factor we considered was the correlation between PET parameters and tumor regression. Thus, 70% SUVmax was chosen as the most suitable SUV threshold for both FLT and FDG PET.

In this study, FLT PET was not superior to FDG PET. In diagnosis, it showed no more visualized information compared with FDG PET. And with regard to the result of Spearman Rank test, FDG PET seemed to have more closely correlation with tumor response of NPC after radiotherapy. Nevertheless, FLT is still a promising measurement, as it is tracer of tumor cell proliferation. Proliferation that occurs during radiotherapy increases the number of target cells and, consequently, the probability of tumour recurrence. Vera *et al*. compared FLT, FDG and Fmiso PET used before and during RT of NSCLC, and found that at 46 Gy, FLT SUVmax values were significantly lower (p < 0.0006) in both tumors and nodes than baseline, suggesting a fast decrease in the proliferation of both tumors and nodes exists during radiotherapy with differences in metabolism (borderline significant decrease) and hypoxia (stable)^37^.

Choosing an optimal time for second PET is a point worth our consideration. Several studies have reported FLT PET might predict tumor response at a quite early point during treatment. A phase II study conducted by Kostakoglu *et al*. found that FLT PET imaging of breast cancer after 1 cycle of NACT weakly predicted pathologically CR^38^. Hong *et al*. analyzed patients of metastatic colorectal cancer (mCRC) receiving FOLFOX chemotherapy (leucovorin, 5-fluorouracil, and oxaliplatin) and conducted FLT PET before, during and after the 1^st^ cycle of FOLFOX. Their results showed FLT flare observed during 5-FU infusion of the 1^st^ cycle was associated with poor treatment response in patients with mCRC^39^. In our study, the second FLT PET was arranged after 2 cycles of NACT, for assessing response to NACT combined with MRI. But this point might not be early enough for FLT PET to show its advantages in early prediction of tumor response over FDG PET.

There was another interesting point in our results. After 2 cycles of NACT, only one patient achieved CR evaluated by MRI, while 4 patients were evaluated as CR by FLT PET and 5 were evaluated as CR by FDG PET. And correlation between post-NACT MRI assessment and tumor regression after radiotherapy was poor, while medium to strong correlation was validated between PET parameters and tumor response after radiotherapy. This means some residual lesion showed by MRI may have low biological activity, and indicates that PET/CT might be of important reference value when delineating gross target volume (GTV) of RT for NPC, while MRI is the main reference now. GTV may be lessened to a smaller volume of proliferative or metabolic activity according to PET/CT. Value of PET/CT in improving delineation of GTV and reducing inter-observer variability has been recognized in various malignant tumors. Because of its accurate target volume delineation and particularly identification of pathologically positive lymph nodes, FDG PET/CT has played an important role in delineating GTV of NSCLC^40^,^41^. And International Atomic Energy Agency (IAEA) has formally recommended to use FDG PET/CT for the purposes of radiotherapy target volume delineation (TVD) for curative intent treatment of NSCLC^42^. Similar use of PET/CT has been applied in prostate and rectal cancer^43^,^44^. In HNC, PET-planning has been confirmed that it translated into smaller GTV, CTV and PTV for the primary tumor volumes, and also demonstrated an improvement on dosimetry by lowering dose to certain organs at risk (OAR)^45^. For NPC RT planning, situation is no easier than HNC, for its deep location and complicated structures of OAR around it. Till now we still lack similar study applying PET/CT in TVD of NPC, but on the basis of all above researches, this idea is believed to be promising. Combination of PET/CT and MRI will bring more advantages in TVD.

Small sample was the limitation of this study, which might be a factor restricting us to revealing advantages of FLT PET over FDG PET in predicting tumor response. On the other hand, because of the relatively well prognosis of NPC, no local recurrence, metastasis or death had been observed by the time of this article. Thus, the prognostic value of FLT and FDG PET were not discussed in this article. Follow-up on these 20 patients will be continued, so as to reveal long-term prognosis and its correlationship with PET parameters. Moreover, prospective studies of NPC with larger samples are expected to compare the efficacy of FLT and FDG PET in predicting tumor response and long-term prognosis.

## Conclusions

Both ^18^F-FDG and ^18^F-FLT PET have the potential to predict tumor regression. FLT PET failed to show advantage over FDG PET according to our preliminary results.

## Additional Information

**How to cite this article**: Qi, S. *et al*.^18^F-FLT and ^18^F-FDG PET/CT in Predicting Response to Chemoradiotherapy in Nasopharyngeal Carcinoma: Preliminary Results. *Sci. Rep.* **7**, 40552; doi: 10.1038/srep40552 (2017).

**Publisher's note:** Springer Nature remains neutral with regard to jurisdictional claims in published maps and institutional affiliations.

## Figures and Tables

**Table 1:** Patient Characteristics.

Characteristic		No. of patients	Percent(%)
Age	Median	49	
	Range	19–64	
Gender	Male	18	90.0
	Female	2	10.0
T stage	T1-2	13	65.0
	T3-4	7	35.0
N stage	N0-1	5	25.0
	N2-3	15	75.0
TNM stage	III	13	65.0
	IV	7	35.0

**Table 2:** Comparison of Maximal Uptake Value between FLT and FDG PET.

Parameters	FLT	FDG	P value
ΣSUVmax	31.01 ± 11.92	48.50 ± 18.65	0.000
SUVmax-T	6.56 ± 2.30	10.61 ± 4.24	0.000
SUVmax-N	7.06 ± 1.89	12.23 ± 4.57	0.000

**Table 3:** Correlation between Post-NACT PET Parameters and Tumor Regression.

FLT				FDG			
Threshold	Value	r	p	Threshold	Value	r	p
	ΣSUVmax	0.602	0.005		ΣSUVmax	0.669	0.001
	Per-SUVmax	−0.456	0.043		Per-SUVmax	**−0.760**	0.000
40%	ΣSUVmean	0.584	0.007	40%	ΣSUVmean	0.687	0.001
	Per-SUVmean	**−0.602**	0.005		Per-SUVmean	−0.779	0.000
	ΣPTV	0.511	0.021		ΣMTV	0.614	0.004
	Per-PTV	−0.456	0.043		Per-MTV	−0.504	0.024
	ΣTLT	0.529	0.016		ΣTLG	0.765	0.000
	Per-TLT	−0.401	0.079		Per-TLG	**−0.801**	0.000
50%	ΣSUVmean	0.602	0.005	50%	ΣSUVmean	0.669	0.001
	Per-SUVmean	**−0.620**	0.004		Per-SUVmean	**−0.779**	0.000
	ΣPTV	0.566	0.009		ΣMTV	0.687	0.001
	Per-PTV	−0.547	0.012		Per-MTV	−0.559	0.010
	ΣTLT	0.584	0.007		ΣTLG	0.724	0.000
	Per-TLT	−0.566	0.009		Per-TLG	−0.724	0.000
60%	ΣSUVmean	0.584	0.007	60%	ΣSUVmean	0.614	0.004
	Per-SUVmean	**−0.620**	0.004		Per-SUVmean	−**0.760**	0.000
	ΣPTV	0.584	0.007		ΣMTV	**0.760**	0.000
	Per-PTV	−0.584	0.007		Per-MTV	−0.632	0.003
	ΣTLT	0.566	0.009		ΣTLG	0.742	0.000
	Per-TLT	−0.547	0.012		Per-TLG	−0.724	0.000
70%	ΣSUVmean	0.602	0.005	70%	ΣSUVmean	0.669	0.001
	Per-SUVmean	**−0.639**	0.002		Per-SUVmean	**−0.760**	0.000
	ΣPTV	0.575	0.008		ΣMTV	**0.760**	0.000
	Per-PTV	−0.529	0.016		Per-MTV	−0.705	0.001
	ΣTLT	0.511	0.021		ΣTLG	0.742	0.000
	Per-TLT	−0.566	0.009		Per-TLG	−0.742	0.000
1.5	ΣSUVmean	0.566	0.009	2.5	ΣSUVmean	0.577	0.008
	Per-SUVmean	−0.566	0.009		Per-SUVmean	−0.705	0.001
	ΣPTV	0.548	0.012		ΣMTV	0.687	0.001
	Per-PTV	**−0.620**	0.004		Per-MTV	−0.687	0.001
	ΣTLT	0.529	0.016		ΣTLG	0.669	0.001
	Per-TLT	−0.566	0.009		Per-TLG	**−0.760**	0.000
